# Mitral Valve Endocarditis with Perforation from a Urinary Source: An Unusual Case and Literature Review

**DOI:** 10.1155/2019/5496851

**Published:** 2019-06-09

**Authors:** Fernando Figueroa Rodriguez, Antonio Faieta Lasarcina, Francisco Davila Grijalva

**Affiliations:** Oakland University Beaumont Hospital, West 13 Mile Road, Royal Oak, MI 48073, USA

## Abstract

*Aerococcus urinae* (AU) is a rare pathogen, identified as gram-positive, catalase-negative coccus that grows in pairs and clusters which has been reported to mainly cause urinary tract infections (UTI), especially in elderly males. Treatment for this microorganism is usually with beta-lactams although cultures with antibiotic susceptibility testing are imperative. We present a case of AU endocarditis initially treated with IV antibiotics; nevertheless, the patient required emergent mitral valve replacement due to severe mitral insufficiency and perforation. We also present an analysis with high-yield points summarizing epidemiology, risk factors, microbiology, clinical features, diagnostic workup, and management of AU in general and AU endocarditis. Finally, we post a literature review of relevant cases and the impact of different variables associated with it.

## 1. Case Presentation

**Figure 1:**
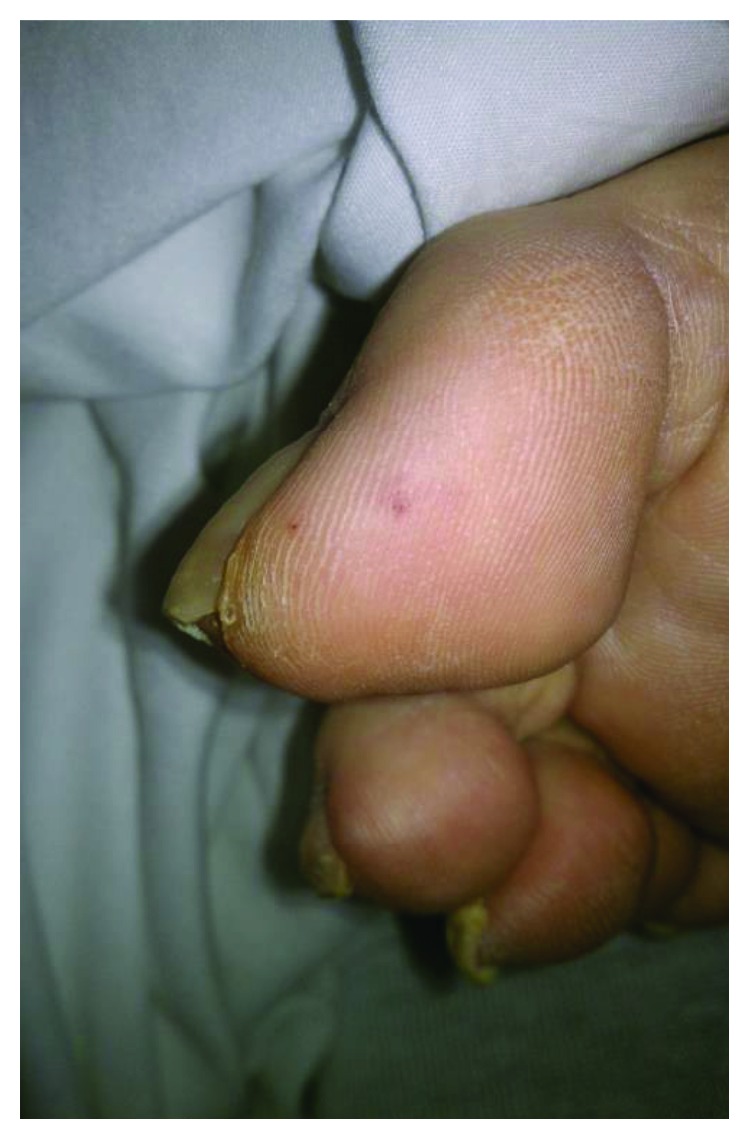
Right first toe with Janeway lesion (vascular emboli phenomena).

**Figure 2:**
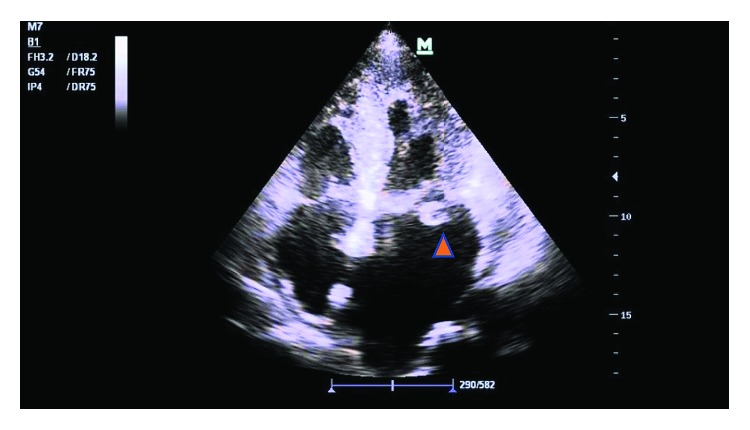
Arrowhead: TTE showing a mobile mass on the posterior leaflet of the mitral valve consistent with a vegetation.

**Figure 3:**
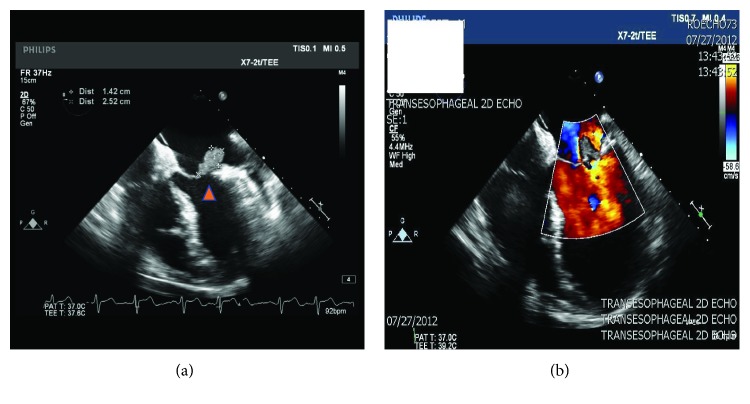
(a) TEE image of vegetation on posterior leaflet of mitral valve, measuring “1.42 cm × 2.52 cm” (arrowhead). (b) TEE image with evidence of blood flow regurgitation with Doppler into the left atrium in the setting of the mitral valve posterior leaflet vegetation.

A 55-year-old male with no significant past medical history presented with a one-week history of nonproductive cough, dyspnea at rest, dysuria, and urinary frequency and urgency. He was diagnosed with a urinary tract infection (UTI) and a viral upper respiratory tract infection by his primary care physician two days prior and was prescribed ciprofloxacin. His symptoms did not improve so he decided to go to the emergency department (ED). Upon presentation, his oxygen saturation was noted to be 90%, with tachycardia and a low-grade fever. On exam, he had significant bibasilar crackles as well as a holosystolic apical murmur; additionally, he was found to have 2+ lower extremity edema and several punctuate macular lesions on his feet, highly suspicious for vascular emboli phenomena (Figure 1). His laboratory studies demonstrated leukocytosis with neutrophilia (14.7 bil/L and 11.3 bil/L, respectively) and evidence of acute kidney injury. The urinalysis bacteriuria and leukocytes. A chest X-ray (CXR) was unremarkable. Blood and urine cultures were obtained, and he was empirically started on ceftriaxone. A transthoracic echocardiogram revealed a mobile mass on the posterior leaflet of the mitral valve consistent with a vegetation. Ejection fraction was calculated to be 40% (Figure 2). Preliminary blood cultures reported gram-positive cocci in pairs and clusters. At day 3, AU was identified as the pathogen in both, blood and urine, cultures. The patient was then started on intravenous gentamicin and penicillin G with plans for a 6-week therapy. He was medically treated for acute heart failure with aggressive diuresis. At day 5, the patient received a transesophageal echocardiogram (TEE) which confirmed a “1.4 cm × 2.5 cm” vegetation attached to the posterior leaflet of mitral valve (Figure 3(a)), with severe mitral regurgitation and posterior leaflet perforation (Figure 3(b)). On day 8, the patient was acutely decompensated, and he was endotracheally intubated due to hypoxic respiratory failure and consequently underwent emergent prosthetic mitral valve replacement. He had a prolonged complicated postoperative period requiring mechanical ventilation, vasopressor support, and hemodialysis. Repeat blood cultures showed resolution of AU bacteremia. Finally, after 3 months, the patient was discharged to a subacute rehabilitation unit.

## 2. Discussion

AU is a rare pathogen, first isolated in 1953 found in lobsters and thought to be of no clinical significance. It was until 1967 when it was first described to cause infection in humans [1]. This organism is a gram-positive, catalase-negative growing coccus in clusters that belongs to a bacterial group referred to as Aerococcus-like organisms (ALO) [2]. It has been associated with UTI mainly, nevertheless there are reports that identify it as the culprit of blood stream infections and endocarditis. Due to difficulties in the biochemical identification, the incidence of infections with this microorganism has likely been underestimated. Currently, it is thought to be the cause of 0.15 to 0.54% of UTIs [1]; additionally, it is responsible for 0.03 to 0.05% of blood stream infections (BSI). There is not enough data regarding the incidence of AU endocarditis as there have been only 45 reports in English literature. Advanced age (>65 years) and underlying urologic conditions are the best-known risk factors for UTI and, potentially, BSI [3] and endocarditis. Both sexes are equally affected [4, 5].

The diagnosis is usually challenging due to the rarity of this pathogen. It is commonly mistaken with Staphylococcus sp. and even Streptococcus viridians; for this reason, molecular diagnostic techniques are often needed to identify it. Testing for leucine aminopeptidase, which is positive only for AU, can be used to differentiate it with Staphylococcus [6]. Secure identification also relies on genetic techniques like 16S ribosomal subunit sequencing or mass spectroscopic methods such as MALDI-TOF [7]. To date, there are no guidelines that recommend starting with any of these modalities, even in the presence of the above risk factors, as they are nonspecific, so the diagnostic algorithms remain the same as for the rest of the pathogens.

Due to the current lack of controlled scientific trials and lack of formalized treatment guidelines, therapy is often empiric and guided by expert opinion. In an effort to standardize a treatment, Yabes et al. in 2018 [8] compiled the reported cases and therapies in literature and found that treatment regimens for AU have largely relied on beta-lactams, with the possible addition of synergistic aminoglycosides for endocarditis. In vitro studies regarding the antibiotic susceptibilities of AU have shown susceptibility to amoxicillin, cefotaxime, ceftriaxone, doxycycline, linezolid, meropenem, penicillin, rifampin, and vancomycin. It had variable resistance to clindamycin, erythromycin, and levofloxacin [9–11]. It is, however, inherently resistant to sulfonamides and fluoroquinolones [10]. The duration of treatment vary from ten days for UTI and phlegmon to 14 to 28 days for BSI and 42 days (6 weeks) for endocarditis [8]. It is important to mention that treatment for the latter very commonly includes surgical intervention as well, but indications are the same as for other etiologies.

Overall mortality of AU infections, including UTI and BSI, has been shown to be equivalent to that of other etiologies. For endocarditis in particular, a higher rate of complications and mortality has been described, with 27% more when compared to other causes. It is thought that this could be associated with an older patient population with multiple other comorbidities, making their surgical profile highly unfavorable, and dependent on medical management only [8].

## 3. Literature Review

Between 2000 and 2012, the incidence of endocarditis in the United States increased from 11 per 100,000 population to 15 per 100,000 population [12, 13]. Nowadays, the precise incidence is difficult to ascertain because case definitions have varied over time between authors and between clinical centers. In addition, the presence of predisposing conditions such as rheumatic heart disease or injection drug use is variable over time and between regions and in low- and high-income countries [14, 15].

A literature search on PubMed, Web of Science, Cochrane, and Google Scholar databases revealed 45 cases of AU endocarditis in English literature (Table 1). We present a summary with a mathematical analysis and calculations involving all reports in an effort to identify patterns regarding risk factors, clinical course, treatment modalities, and prognosis.

### 3.1. General Statistics

The general statistics are as follows:

- 85% (39) of the patients were male and only 15% female (7)
- The average age was 72 years, with the youngest patient being 42 years and the oldest 91 years (2)
- Of the forty patients, only three had no reported associated risk factors or comorbidities (6.5%), 93.5% did have risk factors which are as follows:
  - 69% of the patients had urologic comorbidities while only four patients (9.5%) had an underlying known cardiac condition (one patient with aortic stenosis, one patient with atrial septal defect, and one patient with pacemaker
  - Five patients were also reported to have a nonurologic associated malignancy (11.9%)
  - Only two patients (4.6%) had a different risk factor other than the ones mentioned above, a case with untreated hepatitis C and other case with liver failure (2.3% each)
- Regarding urologic risk factors:
  - Current or recent presence of indwelling urinary catheter was seen in eight patients (19%), benign prostatic hypertrophy was also seen in eight patients (19%)
  - Recurrent UTIs were seen in three patients (6.9%), same goes for urethral strictures (three patients—6.9%)
  - History of cystoscopy was found in two patients (4.6%), phimosis was also reported in two patients only (4.6%)
  - Transurethral resection of the prostate, ureteral stent, and suprapubic catheter were seen with one patient each (2.3% per patient)
- In terms of affected valve: 43.4% (20 patients) had involvement of the mitral valve and the same percentage for aortic valve. One patient, 2.3%, had both valves affected; one patient had aortic and tricuspid valves affected. Finally, one patient (2.3%) had a pacemaker. Affected valves were not reported in 3 patients (6.5%)
- For surgical intervention, in 20 cases, it was not reported whether the patient had surgery or not; for the 26 cases, that it was. Statistics are as follows:
  - 14 patients (53.8%) did not have cardiac valve surgery
  - 12 patients (46.2%) did have cardiac valve surgery
  - 80% of the patients that received surgery survived
- Regarding antibiotic treatment: 98% of the patients received antibiotics. In one patient (2%), treatment was not reported
  - In 100% of the cases, a beta-lactam was used
    1. In 5 patients (11.1%), it was used as a single therapy
    2. In the rest of the patients (91%), it was used in combination: in 38 patients (83%), it was combined with an aminoglycoside; in 2 patients (4.5%), it was used along vancomycin; and in 1 patient (2.2%), it was used in combination with aminoglycoside and rifampin
  - In patients that survived, the duration of beta-lactam ranged from 2 to 12 weeks; meanwhile, aminoglycoside ranged from 1 to 6 weeks. Vancomycin was used for 2 weeks in 1 patient (in the other one duration was not reported), and finally rifampin was used for 6 weeks

### 3.2. Mortality Statistics

The mortality statistics are as follows:

- Overall mortality was 28.2% (13 patients)
  - 92.3% (12) were male and only 1 female
  - 84.6% (11) were over 50 years old (average age: 71 years)
- In terms of risk factors:
  - 69.2% (9) had an underlying urologic condition
  - 1 patient (7.6%) had underlying aortic stenosis, and 1 patient (7.6%) had nonurologic malignancy
  - In 2 patients, the underlying comorbidities were not reported
- Most patients that eventually died did not undergo surgical intervention, only 2 patients (15%) had it
- Regarding affected valve:
  - 46.8% (6 patients) had aortic valve endocarditis
  - 38.4% (5 patients) had mitral valve endocarditis
  - 1 patient (7.6%) had both aortic and tricuspid involvement
  - In 1 patient (7.6%), the affected valve was not reported

### 3.3. Analysis

As seen in previous reports, most of the patients were elderly males. The majority had underlying urologic comorbidities; baseline cardiac anomalies like valvular diseases were very seldom seen. Additionally, mitral and aortic valves were equally affected.

All of the patients received treatment with beta-lactam antibiotics. In most of them, aminoglycosides were used in combination; other less commonly used add-on therapies were vancomycin and rifampin. More than half of the patients did not undergo surgery, as they were deemed as poor surgical candidates or did not meet criteria for intervention. Interestingly enough, 100% of the patients that received beta-lactam as monotherapy in combination with surgery survived.

Aerococcus urinae endocarditis is associated with a 28% mortality from our calculations, deeming a high grade of aggressiveness to this microorganism. It is unclear at this time if this high mortality is due to the bacteria itself or the fact that most of the patients were elderly and had other comorbidities. Lack of surgical intervention in most patients possibly plays a role as 80% of the patients that underwent a surgical procedure survived. An underlying urologic condition was commonly seen in the patients that died, although it was recurrently seen in patients that survived as well. The affected valve does not seem to have an impact on mortality.

## 4. Conclusion

AU is an extremely unusual cause of human disease; it has been described to cause urinary tract infections, blood stream infections, and infective endocarditis. We do believe, however, that its actual incidence is higher than reported. Reliable data regarding clinical presentation, diagnosis, and potential treatments of AU are becoming more available to health-care workers making this a promising era in its management. With this case report and literature review, we aim to add critical data to the current medical literature regarding this rare pathogen. We urge health-care professionals to share related experiences in order to build up literature and treat patients in a more appropriate manner; at this time evidently, randomized studies are univariably defiant as with any rare disorder.

## Figures and Tables

**Table 1:** Summary of clinical characteristics of the forty-five AU endocarditis cases reported in English literature as well as our case.

Case	Age (years)	Gender	Comorbidities or risk factors	Affected valve	Surgery	Antibiotic regimen and duration	Survived?
1 [16]	69	M	Cystoscopy	Aortic	No	Beta-lactam and aminoglycoside for 6 weeks	Yes
2 [17]	54	M	Phimosis	Mitral	Yes	Beta-lactam for 6 weeks	Yes
3 [18]	43	M	Untreated hepatitis C	Aortic	No	Beta-lactam and aminoglycoside for 5 days	No
4 [19]	68	M	Indwelling urinary catheter	Aortic	Yes	Beta-lactam 6 weeks and aminoglycoside for 2 weeks	Yes
5 [20]	80	M	Nonurologic malignancy	Aortic	Yes	Beta-lactam for 2 weeks	Yes
6 [21]	77	M	Benign prostatic hypertrophy	Aortic	No	Beta-lactam and vancomycin; duration not reported	No
7 [22]	68	M	Benign prostatic hypertrophy	Mitral	No	Beta-lactam for 3 weeks; oral aminoglycoside duration not reported	Yes
8 [23]	75	M	Cystoscopy	Aortic	Yes	Beta-lactam and aminoglycoside for 6 weeks	Yes
9 [24]	89	M	Transurethral resection of prostate	Mitral	No	Beta-lactam and aminoglycoside for 7 days	No
10 [25]	81	M	Recurrent UTIs	Aortic	No	Beta-lactam 2 weeks and aminoglycoside for 8 days	No
11 [26]	42	M	None	Aortic	Yes	Beta-lactam and aminoglycoside for 6 weeks	Yes
12 [27]	49	M	Not reported	Aortic	Not reported	Beta-lactam and aminoglycoside; duration not reported	Yes
13 [28]	54	M	Urethral stricture	Tricuspid and aortic	No	Beta-lactam and aminoglycoside; duration not reported	No
14 [29]	69	M	Nonurologic malignancy	Aortic	Yes	Beta-lactam for 12 weeks	Yes
15 [30]	62	M	Benign prostatic hypertrophy	Mitral and aortic	Yes	Beta-lactam and rifampin for 6 weeks and aminoglycoside for 2 weeks	Yes
16 [31]	78	M	Aortic stenosis	Aortic	No	Beta-lactam and aminoglycoside for 10 days	No
17 [32]	74	M	Benign prostatic hypertrophy	Mitral	Yes	Beta-lactam and aminoglycoside for 4 weeks	No
18 [33]	81	M	Benign prostatic hypertrophy	Mitral	No	Beta-lactam and aminoglycoside for 6 weeks	Yes
19 [33]	78	M	Indwelling urinary catheter	Aortic	No	Beta-lactam; duration not reported	No
20 [33]	87	M	Benign prostatic hypertrophy	Mitral	No	Not reported	No
21 [33]	78	F	Ureteral stent	Aortic	No	Beta-lactam and vancomycin for 2 weeks	No
22 [34]	48	M	Atrial septal defect	Mitral	No	Beta-lactam and aminoglycoside; duration not reported	Yes
23 [34]	79	F	Recurrent UTIs	Aortic	No	Beta-lactam and aminoglycoside for 6 weeks	Yes
24 [35]	91	M	Indwelling urinary catheter	Mitral	Not reported	Beta-lactam for 4 weeks and aminoglycoside for 10 days	Yes
25 [35]	91	M	Benign prostatic hypertrophy	Mitral	Not reported	Beta-lactam 4 weeks and aminoglycoside for 10 days	Yes
26 [35]	89	F	Not reported	Mitral	Not reported	Beta-lactam 4 weeks and aminoglycoside for 10 days	Yes
27 [35]	86	M	Urethral stricture	Aortic	Not reported	Beta-lactam 4 weeks and aminoglycoside for 10 days	Yes
28 [35]	83	M	Urethral stricture	Mitral	Not reported	Beta-lactam 4 weeks and aminoglycoside for 10 days	Yes
29 [35]	80	F	Not reported	Mitral	Not reported	Beta-lactam 4 weeks and aminoglycoside for 10 days	Yes
30 [35]	77	M	Not reported	Aortic	Not reported	Beta-lactam for 4 weeks and aminoglycoside for 10 days	Yes
31 [35]	75	M	Benign prostatic hypertrophy	Mitral	Not reported	Beta-lactam for 4 weeks and aminoglycoside for 10 days	Yes
32 [35]	74	M	Suprapubic catheter	Not reported	Not reported	Beta-lactam for 4 weeks and aminoglycoside for 10 days	Yes
33 [35]	65	M	Indwelling urinary catheter	Mitral	Not reported	Beta-lactam for 4 weeks and aminoglycoside for 10 days	Yes
34 [35]	53	M	None	Aortic	Not reported	Beta-lactam 4 weeks and aminoglycoside for 10 days	Yes
35 [35]	49	F	Intermittent urinary catheter	Aortic	Not reported	Beta-lactam for 4 weeks and aminoglycoside for 10 days	Yes
36 [35]	81	M	Indwelling urinary catheter	Mitral	Not reported	Beta-lactam for 4 weeks and aminoglycoside for 10 days	Yes
37 [35]	74	F	Nonurologic malignancy	Not reported	Not reported	Beta-lactam for 4 weeks and aminoglycoside for 10 days	Yes
38 [36]	87	M	Nonurologic malignancy	Mitral	Not reported	Beta-lactam and aminoglycoside; duration not reported	No
39 [36]	77	M	Liver failure	Aortic	Not reported	Beta-lactam and aminoglycoside; duration not reported	Yes
40 [36]	83	M	Indwelling urinary catheter	Mitral	Not reported	Beta-lactam and aminoglycoside; duration not reported	Yes
41 [36]	73	M	Suprapubic catheter	Not reported	Not reported	Beta-lactam and aminoglycoside; duration not reported	No
42 [36]	88	F	Aortic stenosis	Aortic	Not reported	Beta-lactam and aminoglycoside; duration not reported	Yes
43 [8]	43	M	Indwelling urinary catheter	Mitral	Yes	Beta-lactam and aminoglycoside for 6 weeks	Yes
44 [37]	49	M	Phimosis and recurrent UTIs	Mitral	Yes	Beta-lactam and aminoglycoside for 5 days	No
45 [38]	84	M	Pacemaker and nonurologic malignancy	Pacemaker	Yes	Beta-lactam for 6 weeks	Yes
Present case	55	M	None	Mitral	Yes	Beta-lactam and aminoglycoside for 6 weeks	Yes

## References

[1] Higgins A., Garg T. (2017). *Aerococcus urinae*: an emerging cause of urinary tract infection in older adults with multimorbidity and urologic cancer. *Urology Case Reports*.

[2] Williams R. E. O., Hirch A., Cowan S. T. (1953). *Aerococcus*, a new bacterial genus. *Journal of General Microbiology*.

[3] Schuur P. M. H., Kasteren M. E. E. ., Sabbe L., Vos M. C., Janssens M. M. P. C., Buiting A. G. M. (1997). Urinary tract infections with *Aerococcus urinae* in the south of the Netherlands. *European Journal of Clinical Microbiology and Infectious Diseases*.

[4] Senneby E., Petersson A. C., Rasmussen M. (2012). Clinical and microbiological features of bacteraemia with *Aerococcus urinae*. *Clinical Microbiology and Infection*.

[5] Cahill T. J., Prendergast B. D. (2016). Infective endocarditis. *The Lancet*.

[6] Ruoff K. L. (2002). Miscellaneous catalase-negative, gram-positive cocci: emerging opportunists. *Journal of Clinical Microbiology*.

[7] Rasmussen M. (2013). Aerococci and aerococcal infections. *Journal of Infection*.

[8] Yabes J. M., Perdikis S., Graham D. B., Markelz A. (2018). A rare case of Aerococcus urinae infective endocarditis in an atypically young male: case report and review of the literature. *BMC Infectious Diseases*.

[9] Hirzel C., Hirzberger L., Furrer H., Endimiani A. (2016). Bactericidal activity of penicillin, ceftriaxone, gentamicin and daptomycin alone and in combination against *Aerococcus urinae*. *International Journal of Antimicrobial Agents*.

[10] Clinical and Laboratory Standards Institute (2016). *Performance Standards for Antimicrobial Susceptibility Testing*.

[11] Humphries R. M., Hindler J. A. (2014). *In vitro* antimicrobial susceptibility of *Aerococcus urinae*. *Journal of Clinical Microbiology*.

[12] Pant S., Patel N. J., Deshmukh A. (2015). Trends in infective endocarditis incidence, microbiology, and valve replacement in the United States from 2000 to 2011. *Journal of the American College of Cardiology*.

[13] Toyoda N., Chikwe J., Itagaki S., Gelijns A. C., Adams D. H., Egorova N. N. (2017). Trends in infective endocarditis in California and New York State, 1998-2013. *JAMA*.

[14] Tleyjeh I. M., Abdel-Latif A., Rahbi H. (2007). A systematic review of population-based studies of infective endocarditis. *Chest*.

[15] Ambrosioni J., Hernandez-Meneses M., Téllez A. (2017). The changing epidemiology of infective endocarditis in the twenty-first century. *Current Infectious Disease Reports*.

[16] Tathireddy H., Settypalli S., Farrell J. J. (2017). A rare case of Aerococcus urinae infective endocarditis. *Journal of Community Hospital Internal Medicine Perspectives*.

[17] Kotkar K. D., Said S. M., Michelena H., Wanta B., Fritock M. D., Baddour L. M. (2016). Right coronary artery septic embolization secondary to *Aerococcus urinae* native mitral valve endocarditis. *The Annals of Thoracic Surgery*.

[18] Gritsch W., Nagl M., Hausdorfer J., Gschwendtner A., Pechlaner C., Wiedermann C. J. (1999). Septicaemia and endomyocarditis caused by Aerococcus urinae. *Wiener Klinische Wochenschrift*.

[19] Alozie A., Yerebakan C., Westphal B., Steinhoff G., Podbielski A. (2012). Culture-negative infective endocarditis of the aortic valve due to *Aerococcus urinae*: a rare aetiology. *Heart, Lung & Circulation*.

[20] Ho E., Coveliers J., Amsel B. J. (2010). A case of endocarditis due to Aerococcus urinae. *The Journal of Heart Valve Disease*.

[21] Kass M., Toye B., Veinot J. P. (2008). Fatal infective endocarditis due to *Aerococcus urinae*—case report and review of literature. *Cardiovascular Pathology*.

[22] Tekin A., Tekin G., Turunç T., Demiroğlu Z., Kızılkılıç O. (2007). Infective endocarditis and spondylodiscitis in a patient due to *Aerococcus urinae*: first report. *International Journal of Cardiology*.

[23] Ebnother C., Altwegg M., Gottschalk J., Seebach J. D., Kronenberg A. (2002). *Aerococcus urinae* endocarditis: case report and review of the literature. *Infection*.

[24] Schuur P. M. H., Sabbe L., van der Wouw A. J., Montagne G. J., Buiting A. G. M. (1999). Three cases of serious infection caused by *Aerococcus urinae*. *European Journal of Clinical Microbiology and Infectious Diseases*.

[25] Skov R. L., Klarlund M., Thorsen S. (1995). Fatal endocarditis due to *Aerococcus urinae*. *Diagnostic Microbiology and Infectious Disease*.

[26] Gompelman M., Rozemeijer W., Kortmann W. (2014). A life-threatening complication of an ordinary urinary tract infection?. *The Netherlands Journal of Medicine*.

[27] Westmoreland K., Halstead D. C., DuBose P. V. (2014). Infectious endocarditis in 49-year-old man and discussion of phenotypic characteristics of *Aerococcus urinae* and Viridans streptococci. *Laboratory Medicine*.

[28] Siddiqui B., Chaucer B., Chevenon M., Fernandes D., Rana M., Nfonoyim J. (2016). *Aerococcus urinae* associated aortic and tricuspid valve infective endocarditis. *IDCases*.

[29] Slany M., Freiberger T., Pavlik P., Cerny J. (2007). Culture-negative infective endocarditis caused by Aerococcus urinae. *The Journal of Heart Valve Disease*.

[30] Bruegger D., Beiras-Fernandez A., Weis F., Weis M., Kur F. (2009). Extracorporeal support in a patient with cardiogenic shock due to Aerococcus urinae endocarditis. *The Journal of Heart Valve Disease*.

[31] Kristensen B., Nielsen G. (1995). Endocarditis caused by *Aerococcus urinae*, a newly recognized pathogen. *European Journal of Clinical Microbiology and Infectious Diseases*.

[32] Melnick S., Nazir S., Hingorani R., Wexler P. (2016). *Aerococcus urinae*, a rare cause of infective endocarditis. *BML Case Reports*.

[33] de Jong M. F. C., Soetekouw R., ten Kate R. W., Veenendaal D. (2010). *Aerococcus urinae*: severe and fatal bloodstream infections and endocarditis. *Journal of Clinical Microbiology*.

[34] Zbinden R., Santanam P., Hunziker L., Leuzinger B., von Graevenitz A. (1999). Endocarditis due to *Aerococcus urinae*: diagnostic tests, fatty acid composition and killing kinetics. *Infection*.

[35] Sunnerhagen T., Nilson B., Olaison L., Rasmussen M. (2016). Clinical and microbiological features of infective endocarditis caused by aerococci. *Infection*.

[36] Senneby E., Goransson L., Weiber S., Rasmussen M. (2016). A population-based study of aerococcal bacteraemia in the MALDI-TOF MS-era. *European Journal of Clinical Microbiology & Infectious Diseases*.

[37] Adomavicius D., Bock M., Vahl C.-F., Siegel E. (2018). Aerococcus *urinae* mitral valve endocarditis-related stroke: a case report and literature review. *Journal of Investigative Medicine High Impact Case Reports*.

[38] Samuelsson S., Kennergren C., Rasmussen M. (2018). A case of pacemaker endocarditis caused by *Aerococcus urinae*. *Case Reports in Infectious Diseases*.

